# Predicting Yield Strength and Plastic Elongation in Body-Centered Cubic High-Entropy Alloys

**DOI:** 10.3390/ma17174422

**Published:** 2024-09-08

**Authors:** Diego Ibarra Hoyos, Quentin Simmons, Joseph Poon

**Affiliations:** 1 Department of Physics, University of Virginia, Charlottesville, VA 22904, USA; nkt2pd@virginia.edu; 2 Department of Materials Science and Engineering, University of Virginia, Charlottesville, VA 22904, USA

**Keywords:** high-entropy alloys, yield strength prediction, plastic strain prediction, machine learning, d parameter, feature interpretation

## Abstract

We employ machine learning (ML) to predict the yield stress and plastic strain of body-centered cubic (BCC) high-entropy alloys (HEAs) in the compression test. Our machine learning model leverages currently available databases of BCC and BCC+B2 entropy alloys, using feature engineering to capture electronic factors, atomic ordering from mixing enthalpy, and the D parameter related to stacking fault energy. The model achieves low Root Mean Square Errors (RMSE). Utilizing Random Forest Regression (RFR) and Genetic Algorithms for feature selection, our model excels in both predictive accuracy and interpretability. Rigorous 10-fold cross-validation ensures robust generalization. Our discussion delves into feature importance, highlighting key predictors and their impact on mechanical properties. This work provides an important step toward designing high-performance structural high-entropy alloys, providing a powerful tool for predicting mechanical properties and identifying new alloys with superior strength and ductility.

## 1. Introduction

High-entropy alloys (HEAs) are a novel class of materials distinguished by their unique multi-element compositions which deliver exceptional mechanical properties [1,2,3,4]. Unlike traditional alloys that primarily depend on one or two elements, HEAs consist of four or more elements in nearly equiatomic ratios, creating a high entropy of mixing. This elevated entropy is thought to play a key role in stabilizing solid solution phases [5]. The superior properties of HEAs, including enhanced strength, ductility, and resistance to wear and corrosion, make them highly suitable for demanding applications in the aerospace, automotive, and energy sectors [1,2,3,4]. Among the various phases in HEAs, the body-centered cubic (BCC) and BCC+B2 (ordered BCC) phases are particularly noteworthy due to their high strength and moderate ductility, which are retained at high temperatures [2,3,4]. These properties are critical for structural applications, making BCC HEAs the focus of extensive research [2,3,4,6,7,8]. However, designing HEAs with tailored properties requires a profound understanding of the relationships between composition, microstructure, and mechanical behavior. Traditional experimental methods for alloy design are laborious and costly, necessitating efficient computational methods to accelerate discovery and optimization. 

Machine learning (ML) has emerged as an indispensable tool in computational materials science, offering the ability to analyze extensive datasets, identify complex patterns, and predict properties based on input features. Recent studies have demonstrated the efficacy of ML in predicting HEA properties [9,10,11,12,13,14,15,16,17,18]. These studies typically involve the collection of extensive experimental data, the selection of relevant features, and the development of predictive models using various ML algorithms. For instance, Sengupta et al. [17] employed a Neural Network model to predict the yield strength and plasticity of refractory high-entropy alloys (RHEAs), using elemental composition and physics-based properties as features. This study also highlighted the role of uncertainty quantification in enhancing predictive reliability. 

In the present study, we employ a robust ML framework based primarily on Random Forest Regression (RFR) to predict the yield strength $\left(\sigma_{YS}\right)$ and plastic strain $\left(\varepsilon_{f}\right)$ of BCC and BCC+B2 HEAs. Our approach leverages physics-based features, including structural, elastic, electronic, and thermodynamic properties, to capture the intricate factors influencing mechanical properties. We curated a substantial dataset from established sources, focusing on compression data for their consistency and availability. Key features integral to our models include the D parameter $\left(\frac{\gamma_{sf}}{\gamma_{usf}}\right)$, which is the ratio of the surface energy ($\gamma_{s}$) to the unstable stacking fault energy ($\gamma_{usf}$); the Variance of Mixing Enthalpy $\left(\sigma_{\Delta H_{mix}}^{2}\right)$, that quantifies the spread of heterogeneity in the interaction strengths among different atomic pairs in the alloys; and the ratio of testing temperature to melting temperature ($\frac{T_{test}}{T_{melt}}$), representing the softening effect.

We selected Random Forest Regression (RFR) as our primary ML algorithm due to its robustness against overfitting and its capability to handle high-dimensional data. Rigorous hyperparameter optimization and feature selection were conducted using a Genetic Algorithm (GA), ensuring the model’s high accuracy and interpretability. Model validation through repeated 10-fold cross-validation confirmed the reliability of our predictions. Additionally, we investigated feature importance, which enhanced the model’s transparency and understanding of the underlying physical mechanisms. 

This study demonstrates the integration of advanced ML techniques with physics-based feature selection to design high-performance HEAs. Our approach offers a powerful predictive tool for designing alloy compositions with outstanding mechanical properties.

## 2. Materials and Methods

**Figure 1:**
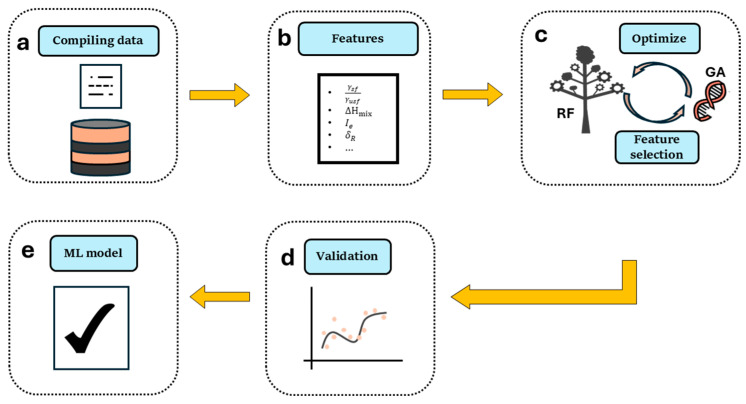
Workflow of the methodological approach. (**a**) Assemble a comprehensive dataset by extracting relevant data from various databases. These data form the foundation for developing the machine learning model. (**b**) Construct a pool of physically based features, including electronic factors, atomic ordering parameters, and other key descriptors relevant to BCC HEAs. These features capture essential aspects of the materials’ properties. (**c**) Optimize the Random Forest Regression (RFR) model by tuning hyperparameters and employing Genetic Algorithms (GA) for feature selection. This step enhances the model’s predictive accuracy and ensures it remains interpretable. (**d**) Validate the model’s performance through rigorous 10-fold cross-validation (CV), ensuring its robustness and generalization to new data. (**e**) The final output is an interpretable machine learning model that provides insights into the most significant features influencing the mechanical properties of BCC HEAs.

This study presents a comprehensive methodological approach based on machine learning to predict the mechanical properties of high-entropy alloys (HEAs), as outlined in Figure 1. Our approach includes several key stages, each contributing to the development and validation of a robust predictive model.

We initiated the process by compiling an extensive dataset using various databases, focusing specifically on compression data for consistency and reliability. This dataset encompasses both single-phase BCC HEAs and BCC+B2 HEAs, with recorded measurements of yield strength $\left(\sigma_{YS}\right)$ and plastic strain $\left(\varepsilon_{f}\right)$. 

A critical component of our methodology is feature engineering. We identified physics-based features representing structural, elastic, electronic, and thermodynamic properties, which are fundamental to the alloy’s mechanical behavior. To enhance model performance, we employed a Genetic Algorithm (GA) for feature selection. This approach allowed us to systematically evaluate different feature subsets, identifying the most relevant features for the Random Forest Regression model. The GA’s evolutionary processes of crossover and mutation were instrumental in refining the feature sets [19]. We conducted a direct analysis of the features selected by the GA to understand their contribution to the model’s predictions. This analysis provided valuable insights into the physical significance of each feature, elucidating their impact on the mechanical properties of HEAs.

The robustness of our model was validated through 10-fold cross-validation (10 CV), ensuring reliable performance and generalizability to new data.

In summary, our methodological framework integrates data collection, feature engineering, model optimization, and feature analysis to construct a reliable machine learning model for predicting the mechanical properties of high-entropy alloys. This approach not only contributes to the advancement of materials science, but also provides practical tools for designing high-performance HEAs.

## 3. Results

### 3.1. Data Compilation and Preprocessing for Random Forest Regression Model

Accurate machine learning models for predicting the mechanical properties of high-entropy alloys (HEAs) require a comprehensive dataset compiled from various established databases [20,21,22]. Our study focused on both single-phase BCC HEAs and BCC+B2 HEA composites to capture a broad spectrum of mechanical behaviors and phase interactions, thus enhancing the robustness and generalizability of our predictive models. We prioritized compression data over tensile data due to their greater availability and consistency, specifically targeting yield strength $\left(\sigma_{YS}\right)$ and plasticity $\left(\varepsilon_{f}\right)$. Tensile properties were excluded as they involve different mechanisms and are less frequently reported for BCC phases.

The dataset comprises alloys subjected to varied processing conditions, including as-cast, annealed, and others, with mechanical testing conducted across a temperature range from 25 °C to 1000 °C. It is important to note that not all alloys were tested across the full temperature range; some of them were evaluated exclusively at ambient conditions. The discrepancy in data volumes between yield strength and plasticity stems from variability in data availability across the databases used. Yield strength is a commonly reported metric, whereas plasticity data are sometimes omitted or reported less frequently, particularly in studies primarily focused on strength. Consequently, our dataset for plasticity is smaller than that for yield strength. This approach resulted in a robust dataset comprising 269 single-phase BCC alloys and 64 BCC+B2 alloys for yield strength, and 153 BCC alloys and 41 BCC+B2 alloys for plasticity.

When multiple values were reported for the same alloy, we averaged these values to mitigate discrepancies and maintain a consistent dataset. Although the dataset is inherently imbalanced, with more data available for BCC alloys, the Random Forest Regression (RFR) model is particularly adept at handling such disparities due to its ensemble nature and flexible structure [23]. This robustness against data imbalances is a significant advantage of RFR, enhancing its reliability for our applications.

Effective data preprocessing is crucial before training the model. In our study, we focused on scaling property-based features to ensure consistency. We employed the StandardScaler, which standardizes features by removing the mean and scaling to unit variance, resulting in each feature having a mean of 0 and a standard deviation of 1. This step ensures that all features contribute equally to the model, and that the training process is not biased by features with larger numerical ranges.

### 3.2. Feature Set Definition, Interpretation, and Analysis

In developing feature sets, we focused exclusively on incorporating physics-based properties derived from the intrinsic physical properties of the individual elements in the alloy. These properties, such as atomic size, Electronegativity, elastic moduli, and others, are systematically combined based on the atomic fractions of the constituent elements in any given alloy composition. This approach allows the physical feature parameters to adapt seamlessly to changes in alloy compositions, ensuring that the model accurately captures composition-dependent behavior. By leveraging this methodology, our model can generalize predictions to HEAs not included in the original compositional dataset, providing valuable insights into the fundamental relationships between material characteristics and their mechanical behavior.

In this study, we identified 28 physics-based features to sufficiently cover structural, elastic, electronic, and thermodynamic properties. These features include the following: Young’s $\left(E\right)$ [24,25], Shear $\left(\mu \right)$ [24,25], and Bulk Modulus $\left(K\right)$ [24,25]; Poisson ratio $\left(\nu \right)$ [24,25]; Radius Mismatch $\left(\delta_{R}\right)$ [26]; Atomic Size Misfit $\left(\varepsilon_{ave}^{4/3}\right)$ [27]; Residual Strain $\left(\epsilon \right)$ [28]; Strain Energy $\left(\frac{E_{2}}{E_{0\;}}\right)$ [26]; Electronegativity $\left(\delta_{\chi }\right)$ [26,29]; Valence Electron Concentration (VEC) [26,29]; Ionization Energy $\left(I_{e}\right)$ [16]; Atomic Planar Density $\left(A_{\rho }\right)$ [16]; Mixing Entropy $\left(\Delta S_{mix}\right)$ [26]; Mixing Enthalpy $\left(\Delta H_{mix}\right)$ [26,30]; and Omega $\left(\Omega \right)$ [26] (details in Supplementary Information). Key features include the D parameter $\left(\frac{\gamma_{sf}}{\gamma_{usf}}\right)$, Variance of Mixing Enthalpy $\left(\sigma_{\Delta H_{mix}}^{2}\right)$, and the Softening $\left(\frac{T_{test}}{T_{melt}}\right)$.

#### 3.2.1. D Parameter ($\frac{\gamma_{sf}}{\gamma_{usf}}$)

The D parameter is a critical metric for predicting the room-temperature ductility of body-centered cubic (BCC) high-entropy alloys (HEAs). This parameter arises from analyzing the competition at the nanoscale between cleavage and dislocation emission at sharp crack tips. Based on an earlier work of Mak et al. [31], Hu et al. [32] proposed that BCC multicomponent alloys with a higher $D=\frac{\gamma_{sf}}{\gamma_{usf}}\;$ ratio are more likely to demonstrate inherent ductility. Here, $\gamma_{usf}$ is the unstable stacking fault energy, and $\gamma_{s}$ is the surface energy. A lower $\gamma_{usf}$ makes it easier to emit dislocations, rendering the alloy to exhibit ductility. By employing first-principles calculations to construct surrogate models, they observed a positive correlation between the D parameter and experimentally observed plasticity across various measurements.

The D parameter must be computed to integrate it into the machine learning models. We utilize the Effective Medium Calculation (EMC) method [33] to reduce computational burden while maintaining fidelity to experimental and Density Functional Theory (DFT)-calculated values. The EMC-computed D parameter is

$$\frac{\gamma_{sf}}{\gamma_{usf}}(D\;parameter)=\frac{\left(\sum_{i}c_{i}*V_{i}*\;\frac{\gamma_{sf_{i}}}{\gamma_{usf_{i}}}\right)}{\sum_{i}c_{i}*V_{i}}$$

where $c_{i}$ and $V_{i}$ are the atomic percentage and volume fraction for the i-th element, respectively, and $\frac{\gamma_{sf_{i}}}{\gamma_{usf_{i}}}$ is the ratio for the i-th element. Accurate performance requires appropriately selecting the atomic planes for the $\gamma_{s}$ and $\gamma_{usf}$ ratios for each constituent element. This selection process is crucial given that HEAs are composed of elements with various intrinsic crystal structures.

The target structure for these HEAs is BCC, so the effective medium theory calculations should reflect properties relevant to the BCC crystal structure. Specifically, the choice of atomic planes for the $\gamma_{s}$ and $\gamma_{usf}$ ratios must align with the planes that are significant for dislocation activities in a BCC lattice. In BCC crystals, the most relevant planes for dislocation activity and stacking faults are the (110) planes, as they are closely packed and are typically the planes along which dislocations move [34]. For surface energy calculations, the (100) plane usually exhibits the lowest surface energy, whereas the (111) plane is more relevant for surface defect and energy considerations; it is the latter that will be used [35].

When dealing with elements that have an intrinsic Face Centered Cubic (FCC) or Hexagonal Close Packed (HCP) structure, it is necessary to map $\gamma_{s}$ and $\gamma_{usf}$ values onto the equivalent BCC planes. $\gamma_{usf}$ values from FCC (111) planes or HCP (0001) planes can serve as proxies for the BCC (110) planes [36]. Similarly, the surface energies of these elements should be mapped to the closest BCC plane equivalents. For FCC elements, the (111) plane can be used as a proxy for the BCC (100) plane due to its low surface energy, and the (100) plane can be mapped to the BCC (111) plane [35]. For HCP elements, the (0001) plane can serve as a proxy for the BCC (100) plane, and the (10-10) plane can be used for the BCC (111) plane [37].

For stacking fault energy calculations, consider dislocation motion along the <111> direction in FCC structures and the <11–20> direction in HCP structures, which can be mapped to the <111> direction in the BCC structure for consistency [33]. For surface energy calculations, directions normal to the chosen planes are used. $\gamma_{s}$ values were extracted from [38]. $\gamma_{usf}$ values were extracted from [39,40,41,42,43,44,45,46].

**Figure 2:**
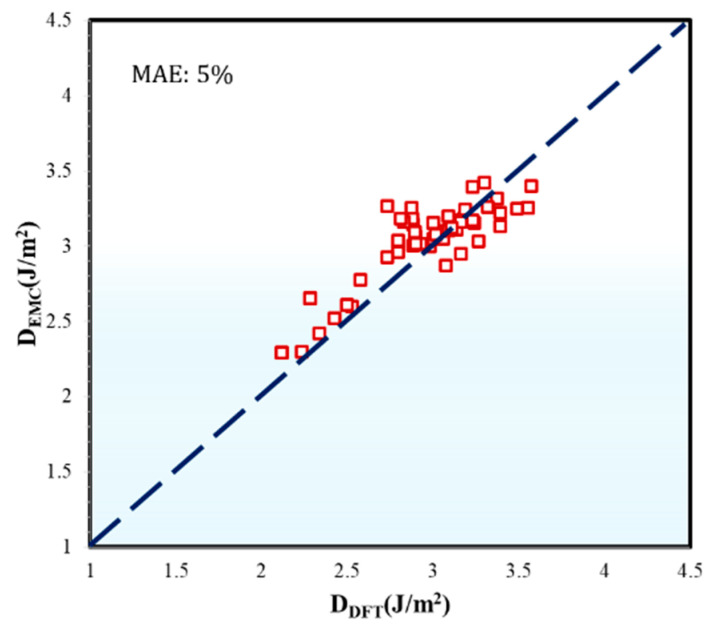
Plot showing the EMC-computed D parameter versus D parameter values obtained from DFT calculations as referenced in [32]. The red squares represent the individual data points for the EMC-computed D parameter. The blue dotted line indicates a perfect linear fit, serving as a reference for the accuracy of the EMC modeling. The Mean Absolute Error of 5% reflects the overall accuracy of the EMC modeling in relation to the DFT values.

By following this approach, the EMC-computed D parameter accurately reflects the dislocation and surface properties pertinent to the BCC structure of the HEAs, while considering the intrinsic crystal structures of the individual constituent elements. This demonstrates strong alignment with DFT-calculated values [32], showing only a 5% Mean Absolute Error (Figure 2).

#### 3.2.2. $\sigma_{\Delta H_{mix}}^{2}$ (Variance of Mixing Enthalpy)

The variance of $\Delta H_{mix}$ quantifies the spread of heterogeneity in the interaction strengths among different atomic pairs in the alloys. It is expressed as

$$\sigma_{\Delta H_{mix}}^{2}=\frac{1}{2}\sum_{i\neq j}^{n}x_{i}{x_{j}\left(H_{ij}-{\Delta H}_{mix}\right)}^{2}$$

High variance indicates significant differences in the interaction strengths between various atomic pairs. This heterogeneity can lead to the formation of composite phases such as BCC+B2 [47]. On the other hand, high variance might also indicate a higher degree of Long-Range Order (LRO), directly affecting the ductility of the alloys. Conversely, low variance indicates more consistent and uniform interaction strengths between atomic pairs, promoting a single phase and suppressing LRO [4]. Given that our database contains both single-phase BCC and dual-phase BCC+B2 alloys, incorporating $\sigma_{\Delta H_{mix}}^{2}$ as a feature allows for effective segmentation. This segmentation aids in developing more robust predictive models.

#### 3.2.3. Softening $\left(\frac{Testing\;Temperature\left(K\right)}{Melting\;Temperature\left(K\right)}\right)$

This ratio indicates how close the testing temperature is to the alloy’s melting temperature. Higher ratios mean the alloy is tested at temperatures closer to its melting point. At these elevated temperatures, the alloy becomes softer and more plastic due to thermal softening and increased atomic mobility, which can facilitate dislocation movement and reduce resistance to deformation [48]. Consequently, this typically results in lower yield strength due to the ease of dislocation movement. On the other hand, this allows the alloy to deform more plastically before fracturing, thereby potentially increasing the plasticity. Because our data encompass alloys tested at both room temperature and above, this parameter is crucial for discerning the effects that are merely due to thermal softening. Understanding this distinction is essential for accurately interpreting the mechanical behavior of the alloys under different thermal conditions.

#### 3.2.4. Variability in Key Material Properties

In addition to the previous features, we chose to calculate the variance of specific properties such as Atomic Planar Density, Ionization Energy, and D parameter; Bulk, Shear, and Young’s Modulus; Poisson ratio, Electronegativity, and VEC, to capture the inherent heterogeneity and distribution of these properties within the alloy. The variance was computed using the following formula:

$$X_{var}=\frac{1}{\sum_{i=1}^{n}c_{i}}\sum_{i=1}^{n}c_{i}{\left(X_{i}-X_{avg}\right)}^{2}$$

where $c_{i}$ is the atomic percentage for the $i_{th}$ element in an n-component system, and $X_{i}$ is the value of the arbitrary property for the $i_{th}$ element.

High variance in features like Atomic Planar Density and Ionization Energy indicates non-uniform atomic packing and bond strengths, respectively, leading to localized weaknesses and reduced mechanical integrity. Variance in D parameter shows variability in dislocation behavior, whereas low variance suggests consistent resistance to dislocation movement, enhancing mechanical properties.

For elastic properties (Bulk Modulus, Shear Modulus, and Young’s Modulus), high variance indicates non-uniform stiffness, resulting in localized deformation under stress, while low variance suggests uniform stiffness, enhancing mechanical performance. VEC and Electronegativity provide insights into the electronic distribution and bonding character, whereas high variance can lead to phase instability and reduced mechanical properties [29]. The Poisson ratio indicates the variability in ductility, with high variance suggesting regions more prone to cracking [49].

By analyzing the variance of these features, we gain a comprehensive understanding of the alloy’s heterogeneity and its impact on yield strength and plasticity. This information is vital for optimizing the design and performance of BCC and B2 high-entropy alloys, allowing for better prediction and enhancement of their mechanical properties.

### 3.3. Model Optimization Using Random Forest Regression

In this study, we focus on the Random Forest Regression (RFR) model to predict the yield strength $\left(\sigma_{YS}\right)$ and plastic strain $\left(\varepsilon_{f}\right)$ of BCC and BCC+B2 high-entropy alloys (HEAs). In addition to its robustness against overfitting, and effectiveness with high-dimensional data, RFR has the ability to model complex, non-linear relationships. This choice is further justified by its ensemble nature, which mitigates the risk of overfitting, a critical factor given the relatively small size of our dataset.

To enhance the performance and generalizability of the RFR model, we meticulously tuned key hyperparameters through a grid search. The parameters optimized included the number of trees, the maximum depth of the trees, the minimum samples required to split a node, the minimum samples required at a leaf node, and the number of features considered for the best split. This careful tuning process ensured the model’s robustness and predictive accuracy.

Feature selection was conducted using a Genetic Algorithm (GA), which is highly effective in optimizing complex, high-dimensional search spaces [19,50]. The GA operates by evolving a population of potential feature subsets through iterative processes of crossover, mutation, and selection. Each feature subset is evaluated based on its performance with the RFR model, using Root Mean Square Error (RMSE) as the fitness criterion. The state variables in this optimization process are the features themselves, which are binary encoded, and the objective is to minimize the negative RMSE across 10-fold cross-validation.

To manage the complexity of the search, constraints such as a maximum of two to fifteen features per subset were applied. Additionally, crossover and mutation probabilities were carefully selected to balance exploration and exploitation within the feature space. The algorithm was allowed to run for a maximum of twenty generations, with an early stopping criterion if no improvement was observed after five generations. See Table S4 for more information.

This GA-driven approach allowed us to tailor the feature sets to the specific requirements of RFR, thereby maximizing its predictive capabilities. Our initial feature set comprised 28 features derived from the elemental properties and compositional characteristics of the alloys. Pearson correlation was first applied to eliminate highly correlated features, after which the GA screened feature subsets ranging from two to fifteen features. This rigorous feature selection process not only enhanced the model’s performance, but also provided valuable insights into the key factors influencing yield strength and plasticity in BCC and BCC+B2 HEAs. Table 1 provides a detailed summary of the GA parameters and the selected features.

The D parameter, which quantifies the competition between dislocation emission and brittle fracture [31,32], emerged as a significant feature for predicting plasticity, as expected. This aligns with its role in capturing the material’s ability to undergo plastic deformation, which is highly sensitive to these competitive mechanisms. However, the D parameter was not selected as an important feature for predicting yield strength. Yield strength is predominantly influenced by factors such as solid solution strengthening, grain boundary effects, and the intrinsic resistance to dislocation initiation, factors more closely associated with the elastic moduli and atomic size mismatch within the alloy. Consequently, for yield strength, the Genetic Algorithm prioritized features directly related to these properties, highlighting the distinct mechanisms that govern yield strength versus those influencing plasticity.

RFR’s effectiveness was contrasted with other models, such as Neural Networks (NN) and Support Vector Regression (SVR). While NNs are powerful in capturing complex, non-linear relationships, they are more prone to overfitting, especially with small datasets, due to their high capacity and numerous parameters. NNs also require extensive tuning and regularization to generalize well, and are often less interpretable than RFR models. SVR can handle non-linear relationships, but may struggle with high-dimensional data. In comparison, RFR offers a balanced solution with its robustness, flexibility, and interpretability, making it particularly suitable for our application [51].

By leveraging the strengths of RFR, including its ability to handle numerous features and interactions without significant preprocessing, we developed a robust and interpretable framework for predicting the mechanical properties of HEAs. This comprehensive approach provides a deeper understanding of the intricate relationships between alloy composition, microstructure, and mechanical behavior, ultimately guiding the selection of the most effective features and hyperparameters for our predictive models.

### 3.4. Model Performance and Validation Using Random Forest Regression

We optimized the RFR model by tuning key hyperparameters through a grid search, ensuring robust and accurate predictions. To assess the generalizability of our RFR model, we employed 10-fold cross-validation (10 CV). This method splits the data into 10-folds, using 9-folds for training and 1-fold for testing, and repeats the process 10 times with each fold serving as the test set once. This comprehensive evaluation ensures the robustness and generalizability of the model’s performance.

**Figure 3:**
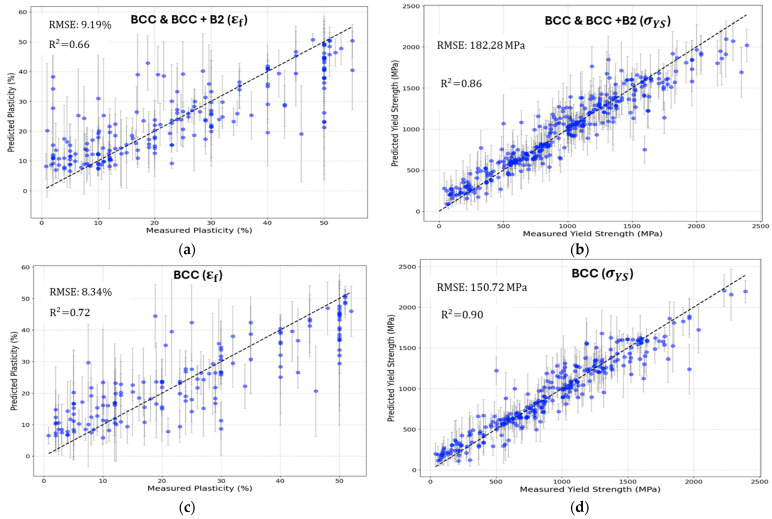
Predicted vs. measured values plotted with error bars representing the prediction uncertainty obtained directly from the Random Forest Regression model during 10 cross-validation with features and hyperparameters optimized. (**a**) Plasticity model using database with BCC and BCC+B2 HEAs, with RMSE 9.19% and R² value of 0.66. (**b**) Yield strength model using database with BCC and BCC+B2 HEAs, with RMSE 182.28MPa and R² value of 0.86. (**c**) Plasticity model using database with only BCC HEAs, with RMSE 8.34% and R² value of 0.72. (**d**) Yield strength model using database with BCC and BCC+B2 HEAs, with RMSE 150.72MPa and R² value of 0.90. Error bars obtained from Random Forest.

Using 10 CV, the RFR model achieved a Root Mean Square Error (RMSE) of 9.19% for plasticity $\left(\varepsilon_{f}\right)$ and 182.28 MPa for yield strength $\left(\sigma_{YS}\right)$. Plots comparing the measured and predicted values, shown in Figure 3, revealed a high R² value of 0.86 for yield strength, indicating a clear linear trend. In contrast, the R² value for plasticity was lower, at 0.66, reflecting greater data dispersion. This discrepancy likely stems from plasticity’s higher sensitivity to microstructural variations introduced during casting and processing, such as grain size and phase distribution, which are less consistent across different studies [4]. Yield strength, being more directly related to dislocation movement and crystal structure, is less affected by these variations.

To improve model accuracy, refining the dataset to include alloys with consistent casting and processing histories could reduce variability, though this must be balanced with maintaining a broad, generalizable dataset. Despite these challenges, the model effectively captures key performance trends, particularly for yield strength.

To further enhance our understanding and improve prediction accuracy, we developed additional models focused exclusively on single-phase BCC HEAs. By eliminating the complexities introduced by dual-phase mechanisms and working with a more homogeneous dataset, these models provided deeper insights into the selected features and yielded an improved performance. The RFR model for single-phase BCC HEAs achieved RMSE of 8.34% for plasticity $\left(\varepsilon_{f}\right)$ and 150.72 MPa for yield strength $\left(\sigma_{YS}\right)$. This more stable dataset, particularly for plasticity, resulted in a clearer linearity at lower measured values.

## 4. Discussion

This study presents a comprehensive machine learning approach to predict the plasticity and yield strength of BCC and BCC+B2 high-entropy alloys (HEAs). By leveraging physics-based features derived from the intrinsic properties of the constituent elements, our models can systematically explore how variations in alloy composition impact key mechanical properties, such as yield strength and plasticity. This capability not only enables the accurate prediction of mechanical behavior, but also facilitates the design of new alloy compositions with optimized properties.

Our models, through the identification and selection of the most influential physical feature parameters, provide valuable insights into elemental combinations that enhance performance. By systematically screening a vast compositional space, we can identify potential high-performance HEAs, streamlining the process of selecting promising candidates for further experimental validation. This approach is more adaptable to new compositional spaces compared to traditional methods relying on elemental composition features, such as those employed by Sengupta et al. (2023) [17].

However, it is essential to acknowledge the strengths and limitations of both approaches to provide a balanced comparison. Our models, developed specifically for BCC and BCC+B2 phases, exhibit robust performance with rigorous validation through repeated 10-fold cross-validation. When compared to the Neural Network (NN) model employed by Sengupta et al., which also aims to predict yield strength and plasticity in refractory high-entropy alloys (RHEAs), our models offer distinct advantages. Specifically, our Random Forest Regression (RFR) model demonstrates lower Root Mean Square Errors (RMSE) when applied to specific single BCC phase or composite BCC+B2 phase alloys, in contrast to the broader scope of RHEAs. While this focus enhances predictive accuracy for BCC and BCC+B2 phases, it may limit the generalizability to other alloy systems, highlighting both an advantage and a potential limitation of our approach.

A key differentiator of our approach is the exclusive use of physics-based features, such as the D parameter, mixing enthalpy variance, and the ratio of testing temperature to melting temperature. These features were selected through Genetic Algorithms, demonstrating their significance as critical factors for mechanical properties. Derived from fundamental principles, these features ensure that our models can make reliable predictions, even for alloy compositions outside of the training database, providing better interpretability for our model. In contrast, the NN model by Sengupta et al. [17] relies on elemental composition features, which can limit its applicability to new compositional spaces. Nonetheless, elemental features can sometimes provide a straightforward representation of material properties, potentially simplifying the model development process.

Neural Networks, while powerful in capturing complex non-linear relationships, are prone to overfitting, particularly with small datasets, due to their high capacity and numerous parameters. This requires extensive tuning and regularization to achieve generalization. Our choice of Random Forest Regression (RFR) addresses this challenge by being robust against overfitting due to its ensemble nature, combining multiple decision trees to enhance predictive accuracy and generalizability. Additionally, RFR models are inherently more interpretable than NNs, making them more suitable for applications where understanding feature importance is crucial. However, it is worth recognizing that NNs, with their ability to model intricate patterns, can sometimes uncover relationships that simpler models might miss.

Sengupta et al. [17] provide a significant contribution by discussing the impact of data quality and errors on model performance, highlighting the importance of uncertainty quantification in enhancing predictive reliability. This is an area where their work excels, providing deeper insights into the uncertainties associated with the predictions. While our validation approach, including 10-fold cross-validation, ensures robust performance across different training set sizes, it does not directly address the quantification of prediction uncertainty to the same extent. Sengupta’s detailed error analysis adds valuable context to the predictive capabilities and reliability of the NN model, underscoring the importance of incorporating uncertainty quantification in future work.

Our use of a Genetic Algorithm (GA) for feature selection enhances the model’s performance by systematically evaluating and selecting the most relevant features. This approach improves predictive accuracy and provides insights into the physical significance of each feature, further validating our model’s interpretability. This interpretability is crucial for understanding the underlying mechanisms governing the mechanical properties of HEAs.

The insights gained from our models can guide the design of new high-performance HEAs. By understanding the role of key features and their interactions, researchers can tailor alloy compositions to achieve desired mechanical properties, thus accelerating the discovery and optimization of advanced materials. Future work could explore the integration of additional physics-based features and the application of more advanced ensemble learning techniques. Expanding the dataset to include more diverse alloy compositions and testing conditions could further enhance the model’s robustness and applicability.

In conclusion, our study demonstrates the effectiveness of a physics-based machine learning approach in predicting the mechanical properties of BCC and BCC+B2 HEAs. By leveraging advanced feature engineering, rigorous validation, and interpretable models, we provide a powerful tool for materials scientists and engineers to design and optimize high-performance alloys. While both our approach and the NN model by Sengupta et al. have their strengths and limitations, the complementary insights they offer underscore the potential of machine learning in advancing materials science.

## Figures and Tables

**Table 1:** Best set of features for RFR selected from GA that best optimize the models for $\varepsilon_{f}$ and $\sigma_{YS}$ of BCC and BCC+B2 HEAs. Not ordered by importance.

$\mathrm{Plastic}\;\mathrm{Strain}\;(\varepsilon_{f}$)	$\mathrm{Yield}\;\mathrm{Strength}\;(\sigma_{YS}$)
${\Delta H}_{mix}$	${\Delta H}_{mix}$
$\frac{T_{test}}{T_{melt}}$	$\frac{T_{test}}{T_{melt}}$
$\sigma_{I_{e}}$	$\sigma_{I_{e}}$
$D(\frac{\gamma_{sf}}{\gamma_{usf}}$)	$\sigma_{\Delta H_{mix}}^{2}$
$\sigma_{D}$	${\Delta S}_{mix}$
ν	$\frac{E_{2}}{E_{0}}$
	$\delta_{R}$
	E
	$\sigma_{E}$

## Data Availability

The data supporting this work are available from the authors upon reasonable request.

## Supplementary Materials

The following supporting information can be downloaded at: https://www.mdpi.com/article/10.3390/ma17174422/s1, Table S1: Definition of physics-based features used in the Machine-Learning Mechanical-Properties model; Table S2: Best set of features for RFR selected from GA that best optimize the models for $\varepsilon_{f}$ and $\sigma_{YS}$ of BCC HEAs; Table S3: Hyperparameters selected for the Random Forest models used to predict Yield Strength and Plasticity in BCC and BCC+B2 High Entropy Alloys (HEAs). The table details the optimized settings for each model, including the number of trees (n_estimators), maximum depth (max_depth), minimum samples split (min_samples_split), minimum samples per leaf (min_samples_leaf), and maximum features (max_features); Table S4: Parameters and details of the Genetic Algorithm (GA) used for feature selection in the Random Forest models predicting Yield Strength and Plasticity in BCC and BCC+B2 High Entropy Alloys (HEAs). The table includes descriptions of the state variables (features), the objective function aimed at minimizing the Negative Root Mean Squared Error (neg_rmse), constraints on the maximum number of features, hyperparameters for the Random Forest Regressor, selection mechanism, crossover and mutation probabilities, stopping criteria, cross-validation method, and the final output indicating the best feature subset and corresponding cross-validation score; Table S5: Dataset comprising Yield Strength values (MPa) for BCC & BCC+B2 HEAs. The dataset includes columns for alloy composition, phase constitution, mechanical testing method, processing condition, testing temperature (°C), and corresponding yield strength values. In this context, “A” denotes alloys that have undergone Annealing, “AC” represents those in the As Cast condition, and "OTHER" refers to all other processing conditions. The mechanical testing method is specified as “C” for Compression testing; Table S6: Dataset comprising Plasticity values (%) for BCC & BCC+B2 HEAs. The dataset includes columns for alloy composition, phase constitution, mechanical testing method, processing condition, testing temperature (°C), and corresponding yield strength values. In this context, “A” denotes alloys that have undergone Annealing, “AC” represents those in the As Cast condition, and “OTHER” refers to all other processing conditions. The mechanical testing method is specified as “C” for Compression testing.
