# Supplementary material for: Predicting Yield Strength and Plastic Elongation in Body-Centered Cubic High-Entropy Alloys
Source: Materials (Basel). 2024 Sep 8;17(17):4422. doi: 10.3390/ma17174422 (PMC11396727; doi:10.3390/ma17174422)
Supplement: Supplementary file 1 [file materials-17-04422-s001.zip › materials-3163686-supplementary.pdf]

Supplementary Information

Diego Ibarra, Quentin Simmons, S. Joseph Poon. Predicting Mechanical Properties in Body-Centered-Cubic High-Entropy Alloys. 2024

Table S1. Definition of physics-based features used in the Machine-Learning Mechanical- Properties model.

**Features**	**Details**
$\frac{\gamma_{sf}}{\gamma_{usf}}$ (D Parameter) $\equiv \frac{\sum_i c_i * V_i * \frac{\gamma_{sf\_i}}{\gamma_{usf\_i}}}{\sum_i c_i * V_i}$	c_i: Atomic percentage for the i_{th}element in an n-component system. (Definitions of n and c_i are the same elsewhere.)<br>V_i: Volume fraction for the i_{th}element<br>γ_{sf_i}: Surface energy for the i_{th} element [38]<br>γ_{usf_i}: Unstable stacking fault energy for the i_{th} element [39-46].
μ (Shear Modulus) $\equiv \sum_{i=1}^{n} \frac{1}{\sum_{i=1}^{n} c_i * \frac{1}{\mu_i}}$<br>[24,25]	μ_i: Shear modulus of the i_{th} element
E (Young's Modulus) $\equiv \sum_{i=1}^{n} \frac{1}{\sum_{i=1}^{n} c_i * \frac{1}{E_i}}$<br>[24,25]	E_i: Young's modulus of the i_{th} element
K (Bulk Modulus) $\equiv \sum_{i=1}^{n} \frac{1}{\sum_{i=1}^{n} c_i * \frac{1}{K_i}}$ [24,25]	K_i: Bulks modulus of the i_{th} element
$\bar{\nu}$ (Poisson's Ratio) $\equiv \sum_{i=1}^{n} c_i * \nu_i$ [24,25]	ν_i: Poisson's ratio of the i_{th} element
VEC $\equiv \sum_{i=1}^{n} c_i * VEC_i$ [26,29]	VEC_i: Valence Electron Count of the i_{th} element
$\Delta H_{mix} \equiv \sum_{i=1}^{n} 4\, \Delta H_{i,j}^{mix}\, c_i c_j$ [26,30]	$\Delta H_{i,j}^{mix}$: The binary mixing enthalpy obtained from the Miedema's model [14] of the i-j element pair.
$\Delta S_{mix} = -R \sum_{i=1}^{N} c_i \ln(c_i)$ [26]	R: The gas constant.

$\Omega \equiv \mathbf{T_m \Delta S_{mix} / \lvert \Delta H_{mix} \rvert}$ [26]	T_m: Alloy melting temperature.
$\varepsilon_{\mathbf{ave}}^{\mathbf{4/3}}$ (Average atomic size misfit) ≡ $\sum_{ij} \left\lvert \frac{1}{a} \frac{da}{dx_i^j} \right\rvert \mathbf{c_i c_j}$ [27]	a = Average lattice constant. $\frac{da}{dx_i^j}$ = Variation of the lattice constant when a small amount of element, i, in the average matrix of the alloys is replaced by an element, j.
$\boldsymbol{\delta_\chi}$ (Electronegativity mismatch) ≡ $\frac{\sum_{i=1}^{n} \sum_{j=1, i \neq j}^{n} c_i c_j * \lvert \frac{\chi_i - \chi}{\chi} \rvert}{\sum_{i=1}^{n} \sum_{j=1, i \neq j}^{n} c_i c_j}$ [26,29]	χ_i: Electronegativity of the i_{th} element. $\chi = \sum_{i=1}^{n} c_i \chi_i$: Average electronegativity.
$\boldsymbol{I_e}$ (Ionization Energy) ≡ $\frac{1}{\sum_{i=1}^{n} c_i} \sum_{i=1}^{n} \boldsymbol{c_i I_{e_i}}$ [16]	I_{e_i}: Ionization Energy of the i_{th} element.
$\boldsymbol{A_\rho}$ (Atomic Planar Density) ≡ $\frac{1}{\sum_{i=1}^{n} c_i} \sum_{i=1}^{n} \boldsymbol{c_i A_{\rho_i}}$ [16]	A_{ρ_i}: Atomic Planar Density of the i_{th} element.
E_2/E_0 (Geometric Strain) ≡ $\sum_{j \geq i}^{n} \frac{c_i * c_j * \lvert r_i + r_j - 2r \rvert^2}{(2r)^2}$ [26]	$r = \sum_{i=1}^{n} c_i r_i$: Average atomic radius.
$\boldsymbol{\delta_R}$(Radius Mismatch) ≡ $\boldsymbol{\delta_R = c_i c_j \lvert r_i - r_j \rvert}$ [26]	$\delta_R = c_i c_j \lvert r_i - r_j \rvert$
$\sqrt{\langle \boldsymbol{\varepsilon}^2 \rangle} = \sum_{i=1}^{N} \mathbf{c_i} \boldsymbol{\varepsilon_i^2}$ Residual Strain [28]	$\varepsilon_i = \frac{\sum_{j=1}^{N} \omega_{ij} c_j}{\sum_{k=1}^{N} A_{ik} c_k} - \frac{4\pi \eta_{ideal}}{N_i \sum_{k=1}^{N} A_{ik} c_k}$: Residual strain of the i-th element in a N-component system $\omega_{ij} = 2\pi \left[1 - \frac{\sqrt{r_i (r_i + 2r_j)}}{r_i + 2r_j} \right]$: Solid angle subtended by the j-th element around the i-th element.

	$A_{ij} = \frac{2\pi x_{ik}}{(x_{ik}+1)^2\sqrt{x_{ik}(x_{ik}+2)}}$: dimensionless constant for the i-th and k-th elements<br>$\eta_{ideal} = \frac{1}{2}\sum_{i=1}^{N}\sum_{j=1}^{N} c_j c_i N_i \left[1 - \frac{\sqrt{x_{ij}(x_{ij}+2)}}{x_{ij}+1}\right]$: ideal atomic packing fraction<br>$x_{ij} = r_i/r_j$: atomic radius ratio<br>N_i: coordinate number of the i-th atom.

Table S2. Best set of features for RFR selected from GA that best optimize the models for ε_f and σ_{YS} of BCC HEAs

Random Forest Regressor	
**Plastic Strain (ε_f)**	**Yield Strength (σ_{YS})**
$\sigma_{\Delta H_{mix}}$	ΔH_{mix}
$\frac{T_{test}}{T_{melt}}$	$\frac{T_{test}}{T_{melt}}$
$D(\frac{\gamma_{sf}}{\gamma_{usf}})$	σ_{I_e}
σ_{I_e}	$\sigma^2_{\Delta H_{mix}}$
A_ρ	ΔS_{mix}
σ_μ	$\frac{E_2}{E_0}$
	δ_R
	E
	σ_E

Table S3. Hyperparameters selected for the Random Forest models used to predict Yield Strength and Plasticity in BCC and BCC+B2 High Entropy Alloys (HEAs). The table details the optimized settings for each model, including the number of trees (n_estimators), maximum depth (max_depth), minimum samples split (min_samples_split), minimum samples per leaf (min_samples_leaf), and maximum features (max_features).

Random Forest Regressor Hyperparameters				
**Hyperparameters**	**Number of trees** Considered: (10,50,100,1000)	**Maximum depth of the trees** Considered : range(3,8)	**Minimum samples required to split a node** Considered : range(1,5)	**Minimum samples required at a leaf node** Considered : range(1,5)
BCC+B2 Plasticity	Selected: 10	Selected: 6	Selected: 2	Selected: 1
BCC+B2 Yield Strength	Selected: 100	Selected: 7	Selected: 2	Selected: 2
BCC Plasticity	Selected: 100	Selected: 7	Selected: 3	Selected: 1
BCC Yield Strength	Selected: 50	Selected: 7	Selected: 3	Selected: 3

Table S4. Parameters and details of the Genetic Algorithm (GA) used for feature selection in the Random Forest models predicting Yield Strength and Plasticity in BCC and BCC+B2 High Entropy Alloys (HEAs). The table includes descriptions of the state variables (features), the objective function aimed at minimizing the Negative Root Mean Squared Error (neg_rmse), constraints on the maximum number of features, hyperparameters for the Random Forest Regressor, selection mechanism, crossover and mutation probabilities, stopping criteria, cross-validation method, and the final output indicating the best feature subset and corresponding cross-validation score.

Genetic Algorithm	
**Parameter**	**Description**
**State Variables**	**Features**: Various physics-based and elemental properties of the material system. The state variables in our GA-based feature selection are the features themselves, which are binary encoded. Each individual in the population represents a potential subset of features, where '1' indicates the inclusion of a feature and '0' indicates its exclusion.
**Objective Function**	Minimize the Negative Root Mean Squared Error for the Random Forest Regressor (RFR) model's performance during cross-validation.

**Constraints**	**Maximum Features**: The algorithm allows a maximum of 2 to 15 features in the feature subset.<br>**Hyperparameters for RFR**: Tuned using GridSearchCV to find the best max_depth, min_samples_split, min_samples_leaf, and n_estimators.
**Selection Mechanism**	Tournament Selection with a tournament size of 3.
**Crossover**	**Probability**: 0.5 (The probability of crossover between pairs of individuals)<br>**Independent Probability**: 0.5 (The probability of each feature being swapped independently during crossover)
**Mutation**	**Probability**: 0.1 (The probability of mutation for an individual)<br>**Independent Probability**: 0.05 (The probability of each feature being independently mutated)
**Stopping Criteria**	**Generations**: The algorithm runs for a maximum of 20 generations.<br>**No Improvement**: The algorithm stops early if there is no improvement in the best score after 5 generations
**Cross-Validation**	10-Fold Cross-Validation (CV=10) is used for evaluating the performance of each feature subset.
**Final Output**	**Best Feature Subset**: The subset of features that resulted in the lowest neg_rmse score.<br>**Best CV Score**: The lowest neg_rmse achieved across all generations.

Table S5. Dataset comprising Yield Strength values (MPa) for BCC & BCC+B2 HEAs. The dataset includes columns for alloy composition, phase constitution, mechanical testing method, processing condition, testing temperature (°C), and corresponding yield strength values. In this context, "A" denotes alloys that have undergone Annealing, "AC" represents those in the As Cast condition, and "OTHER" refers to all other processing conditions. The mechanical testing method is specified as "C" for Compression testing.

**BCC & BCC+B2 Yield Strength Dataset**					
**Alloy**	**Phases**	**Mechanical Testing**	**Processing Condition**	**Test Temperature C**	**Yield Strength (MPa)**
Al0.214Nb0.714Ta0.571Ti1V0.143Zr0.929	BCC	C	OTHER	25.0	1965.0
Al0.214Nb0.714Ta0.571Ti1V0.143Zr0.929	BCC	C	OTHER	800.0	678.0
Al0.214Nb0.714Ta0.571Ti1V0.143Zr0.929	BCC	C	OTHER	1000.0	166.0
Al0.214Nb0.714Ta0.714Ti1Zr0.929	BCC	C	OTHER	25.0	1965.0
Al0.214Nb0.714Ta0.714Ti1Zr0.929	BCC	C	OTHER	800.0	362.0
Al0.214Nb0.714Ta0.714Ti1Zr0.929	BCC	C	OTHER	1000.0	236.0
Al0.25Co1Cr1Cu1Fe1Mn1Ni1Ti1V1	BCC	C	AC	25.0	1465.0

Al0.25Cr0.5Nb0.5Ti1V0.25	BCC	C	AC	25.0	1240.0
Al0.25Mo1Nb1Ti1V1	BCC	C	AC	25.0	1250.0
Al0.25Nb1Ta1Ti1V1	BCC	C	AC	25.0	1330.0
Al0.2Mo1Ta1Ti1V1	BCC	C	AC	25.0	1021.0
Al0.333Nb0.667Ta0.533Ti1V0.133Zr0.667	BCC	C	OTHER	25.0	2035.0
Al0.333Nb0.667Ta0.533Ti1V0.133Zr0.667	BCC	C	OTHER	800.0	796.0
Al0.333Nb0.667Ta0.533Ti1V0.133Zr0.667	BCC	C	OTHER	1000.0	220.0
Al0.3Hf1Nb1Ta1Ti1Zr1	BCC	C	AC	25.0	1188.0
Al0.3Nb1Ta0.8Ti1.4V0.2Zr1.3	BCC	C	OTHER	25.0	1965.0
Al0.3Nb1Ta0.8Ti1.4V0.2Zr1.3	BCC	C	OTHER	800.0	678.0
Al0.3Nb1Ta0.8Ti1.4V0.2Zr1.3	BCC	C	OTHER	1000.0	166.0
Al0.4Hf0.6Nb1Ta1Ti1Zr1	BCC	C	OTHER	25.0	1841.0
Al0.4Hf0.6Nb1Ta1Ti1Zr1	BCC	C	OTHER	800.0	796.0
Al0.4Hf0.6Nb1Ta1Ti1Zr1	BCC	C	OTHER	1000.0	298.0
Al0.4Hf0.6Nb1Ta1Ti1Zr1	BCC	C	OTHER	1200.0	89.0
Al0.5Cr1Nb1Ti2V0.5	BCC	C	AC	25.0	1240.0
Al0.5Hf1Nb1Ta1Ti1Zr1	BCC	C	AC	25.0	1302.0
Al0.5Mo1Nb1Ti1V1	BCC	C	AC	25.0	1625.0
Al0.5Nb1Ta1Ti1V1	BCC	C	AC	25.0	1014.0
Al0.667Nb1Ta0.333Ti1Zr0.333	BCC	C	OTHER	25.0	1280.0
Al0.667Nb1Ta0.333Ti1Zr0.333	BCC	C	OTHER	800.0	728.0
Al0.667Nb1Ta0.333Ti1Zr0.333	BCC	C	OTHER	1000.0	403.0
Al0.6Mo1Ta1Ti1V1	BCC	C	AC	25.0	962.0
Al0.75Hf1Nb1Ta1Ti1Zr1	BCC	C	AC	25.0	1415.0
Al0.75Mo1Nb1Ti1V1	BCC	C	AC	25.0	1260.0
Al1.5Mo1Nb1Ti1V1	BCC	C	AC	25.0	500.0
Al1Co0.5Cr0.5Cu0.5Fe0.5Ni0.5	BCC	C	AC	25.0	1620.0
Al1Co0.5Cr0.5Cu0.5Fe0.5Ni0.5	BCC	C	AC	500.0	1120.0
Al1Co0.5Cr0.5Cu0.5Fe0.5Ni0.5	BCC	C	AC	600.0	805.0
Al1Co0.5Cr0.5Cu0.5Fe0.5Ni0.5	BCC	C	AC	700.0	567.0
Al1Co0.5Cr0.5Cu0.5Fe0.5Ni0.5	BCC	C	AC	800.0	302.0
Al1Co0.5Cr0.5Cu0.5Fe0.5Ni0.5	BCC	C	AC	900.0	214.0
Al1Co0.5Cr0.5Cu0.5Fe0.5Ni0.5	BCC	C	AC	1000.0	116.0
Al1Co0.5Cr0.5Cu0.5Fe0.5Ni0.5	BCC	C	AC	1100.0	79.0
Al1Co1Cr1Fe1Mo0.1Ni1	BCC	C	AC	25.0	1804.0

Al1Co1Cr1Fe1Nb0.1Ni1	BCC	C	AC	25.0	1641.0
Al1Co1Cr1Fe1Ni1	BCC	C	AC	25.0	1184.6
Al1Co1Fe1Ni1	BCC	C	AC	25.0	964.0
Al1Cr0.5Nb1Ti1V1	BCC	C	A	25.0	1300.0
Al1Cr0.5Nb1Ti1V1	BCC	C	A	600.0	1005.0
Al1Cr0.5Nb1Ti1V1	BCC	C	A	800.0	640.0
Al1Cr0.5Nb1Ti1V1	BCC	C	A	1000.0	40.0
Al1Cr1Mo1Nb1Ti1	BCC	C	AC	400.0	1080.0
Al1Cr1Mo1Nb1Ti1	BCC	C	AC	600.0	1060.0
Al1Cr1Mo1Nb1Ti1	BCC	C	AC	800.0	860.0
Al1Cr1Mo1Nb1Ti1	BCC	C	AC	1000.0	594.0
Al1Cr1Mo1Nb1Ti1	BCC	C	AC	1200.0	105.0
Al1Cr1Mo1Ti1	BCC	C	A	25.0	1100.0
Al1Cr1Mo1Ti1	BCC	C	A	400.0	1070.0
Al1Cr1Mo1Ti1	BCC	C	A	600.0	1020.0
Al1Cr1Mo1Ti1	BCC	C	A	800.0	875.0
Al1Cr1Mo1Ti1	BCC	C	A	1000.0	375.0
Al1Cr1Mo1Ti1	BCC	C	A	1200.0	100.0
Al1Mo1Nb1Ti1	BCC	C	A	25.0	1100.0
Al1Mo1Nb1Ti1	BCC	C	A	600.0	520.0
Al1Mo1Nb1Ti1	BCC	C	A	800.0	500.0
Al1Mo1Nb1Ti1	BCC	C	A	1000.0	540.0
Al1Mo1Nb1Ti1	BCC	C	A	1200.0	200.0
Al1Mo1Nb1Ti1V1	BCC	C	AC	25.0	1375.0
Al1Nb1.5Ta0.5Ti1.5Zr0.5	BCC	C	OTHER	25.0	1280.0
Al1Nb1.5Ta0.5Ti1.5Zr0.5	BCC	C	OTHER	800.0	728.0
Al1Nb1.5Ta0.5Ti1.5Zr0.5	BCC	C	OTHER	1000.0	403.0
Al1Nb1Ta1Ti1	BCC	C	AC	25.0	1152.0
Al1Nb1Ta1Ti1	BCC	C	AC	100.0	740.0
Al1Nb1Ta1Ti1	BCC	C	AC	200.0	740.0
Al1Nb1Ta1Ti1V1	BCC	C	AC	25.0	993.0
Al1Nb1Ti1V1	BCC	C	A	25.0	1010.0
Al1Nb1Ti1V1	BCC	C	A	600.0	795.0
Al1Nb1Ti1V1	BCC	C	A	800.0	622.5
Al1Nb1Ti1V1	BCC	C	A	1000.0	134.0

Cr1Mo1Nb1Ti1	BCC	C	A	25.0	1630.0
Cr1Mo1Nb1Ti1	BCC	C	A	200.0	1268.0
Cr1Mo1Nb1Ti1	BCC	C	A	400.0	1115.0
Cr1Mo1Nb1Ti1	BCC	C	A	600.0	1062.0
Cr1Mo1Nb1Ti1	BCC	C	A	800.0	1058.0
Hf0.25Nb0.125Ti1V0.5Zr0.5	BCC	C	AC	25.0	1115.0
Hf0.25Nb0.25Ti1V0.5Zr0.5	BCC	C	AC	25.0	1065.0
Hf0.25Nb0.25Ti1V0.5Zr0.5	BCC	C	AC	600.0	718.0
Hf0.25Nb0.25Ti1V0.5Zr0.5	BCC	C	AC	800.0	135.0
Hf0.25Nb0.375Ti1V0.5Zr0.5	BCC	C	AC	25.0	1025.0
Hf0.25Nb0.5Ti1V0.5Zr0.5	BCC	C	AC	25.0	980.0
Hf0.25Nb0.5Ti1V0.5Zr0.5	BCC	C	AC	600.0	859.0
Hf0.25Nb0.5Ti1V0.5Zr0.5	BCC	C	AC	800.0	195.0
Hf0.25Ti1V0.5Zr0.5	BCC	C	AC	25.0	1160.0
Hf0.25Ti1V0.5Zr0.5	BCC	C	AC	600.0	405.0
Hf0.25Ti1V0.5Zr0.5	BCC	C	AC	800.0	85.0
Hf0.26Nb1Ta1Ti0.578Zr0.416	BCC	C	AC	25.0	845.0
Hf0.26Nb1Ta1Ti0.578Zr0.416	BCC	C	AC	60.0	795.0
Hf0.26Nb1Ta1Ti0.578Zr0.416	BCC	C	AC	100.0	765.0
Hf0.26Nb1Ta1Ti0.578Zr0.416	BCC	C	AC	200.0	650.0
Hf0.26Nb1Ta1Ti0.578Zr0.416	BCC	C	AC	300.0	590.0
Hf0.4Nb1.54Ta1.54Ti0.89Zr0.64	BCC	C	AC	20.0	822.0
Hf0.4Nb1.54Ta1.54Ti0.89Zr0.64	BCC	C	AC	60.0	795.0
Hf0.4Nb1.54Ta1.54Ti0.89Zr0.64	BCC	C	AC	100.0	765.0
Hf0.4Nb1.54Ta1.54Ti0.89Zr0.64	BCC	C	AC	200.0	650.0
Hf0.4Nb1.54Ta1.54Ti0.89Zr0.64	BCC	C	AC	300.0	590.0
Hf0.5Mo0.5Nb1Ti1Zr1	BCC	C	AC	25.0	1176.0
Hf0.5Nb0.5Ta0.5Ti1.5Zr1	BCC	C	AC	25.0	903.0
Hf0.5Nb0.667Ta0.333Ti1Zr0.833	BCC	C	OTHER	-268.8	2283.333333333333
Hf0.5Nb0.667Ta0.333Ti1Zr0.833	BCC	C	OTHER	-196.0	1850.0
Hf0.5Nb0.667Ta0.333Ti1Zr0.833	BCC	C	OTHER	-153.0	1520.0
Hf0.5Nb0.667Ta0.333Ti1Zr0.833	BCC	C	OTHER	-103.0	1333.333333333333
Hf0.5Nb0.667Ta0.333Ti1Zr0.833	BCC	C	OTHER	-43.0	1133.333333333333
Hf0.5Nb0.667Ta0.333Ti1Zr0.833	BCC	C	OTHER	25.0	1046.666666666667
Hf0.5Nb0.667Ta0.333Ti1Zr0.833	BCC	C	OTHER	72.0	900.0

Hf0.75Nb1Ta0.5Ti1.5Zr1.25	BCC	C	OTHER	-268.8	2230.0
Hf0.75Nb1Ta0.5Ti1.5Zr1.25	BCC	C	OTHER	-196.0	1815.0
Hf0.75Nb1Ta0.5Ti1.5Zr1.25	BCC	C	OTHER	-153.0	1595.0
Hf0.75Nb1Ta0.5Ti1.5Zr1.25	BCC	C	OTHER	-103.0	1375.0
Hf0.75Nb1Ta0.5Ti1.5Zr1.25	BCC	C	OTHER	-43.0	1190.0
Hf0.75Nb1Ta0.5Ti1.5Zr1.25	BCC	C	OTHER	25.0	1125.0
Hf0.75Nb1Ta0.5Ti1.5Zr1.25	BCC	C	OTHER	72.0	1030.0
Hf0.75Nb1Ta0.5Ti1.5Zr1.25	BCC	C	OTHER	-268.8	2390.0
Hf0.75Nb1Ta0.5Ti1.5Zr1.25	BCC	C	OTHER	-196.0	1920.0
Hf0.75Nb1Ta0.5Ti1.5Zr1.25	BCC	C	OTHER	-153.0	1370.0
Hf0.75Nb1Ta0.5Ti1.5Zr1.25	BCC	C	OTHER	-103.0	1250.0
Hf0.75Nb1Ta0.5Ti1.5Zr1.25	BCC	C	OTHER	-43.0	1020.0
Hf0.75Nb1Ta0.5Ti1.5Zr1.25	BCC	C	OTHER	25.0	890.0
Hf0.75Nb1Ta0.5Ti1.5Zr1.25	BCC	C	OTHER	72.0	640.0
Hf1Mo0.25Nb1Ta1Ti1Zr1	BCC	C	AC	25.0	1112.0
Hf1Mo0.5Nb1Ta1Ti1Zr1	BCC	C	AC	25.0	1317.0
Hf1Mo0.5Nb1Ti1V0.5	BCC	C	AC	25.0	1260.0
Hf1Mo0.5Nb1Ti1V0.5	BCC	C	AC	1000.0	368.0
Hf1Mo0.5Nb1Ti1V0.5	BCC	C	AC	1200.0	60.0
Hf1Mo0.75Nb1Ta1Ti1Zr1	BCC	C	AC	25.0	1373.0
Hf1Mo1Nb1Ta1Ti1	BCC	C	AC	25.0	1369.0
Hf1Mo1Nb1Ta1Ti1	BCC	C	AC	800.0	822.0
Hf1Mo1Nb1Ta1Ti1	BCC	C	AC	1000.0	778.0
Hf1Mo1Nb1Ta1Ti1	BCC	C	AC	1200.0	699.0
Hf1Mo1Nb1Ta1Ti1	BCC	C	AC	1400.0	367.0
Hf1Mo1Nb1Ta1Ti1Zr1	BCC	C	AC	25.0	1512.0
Hf1Mo1Nb1Ta1Ti1Zr1	BCC	C	AC	800.0	1007.0
Hf1Mo1Nb1Ta1Ti1Zr1	BCC	C	AC	1000.0	814.0
Hf1Mo1Nb1Ta1Ti1Zr1	BCC	C	AC	1200.0	556.0
Hf1Mo1Nb1Ta1Zr1	BCC	C	AC	25.0	1524.0
Hf1Mo1Nb1Ta1Zr1	BCC	C	AC	800.0	1005.0
Hf1Mo1Nb1Ta1Zr1	BCC	C	AC	1000.0	927.0
Hf1Mo1Nb1Ta1Zr1	BCC	C	AC	1200.0	694.0
Hf1Mo1Nb1Ta1Zr1	BCC	C	AC	1400.0	278.0
Hf1Mo1Nb1Ti1Zr1	BCC	C	A	25.0	1575.0

Hf1Mo1Nb1Ti1Zr1	BCC	C	A	800.0	825.0
Hf1Mo1Nb1Ti1Zr1	BCC	C	A	1000.0	635.0
Hf1Mo1Nb1Ti1Zr1	BCC	C	A	1200.0	187.0
Hf1Mo1Ta1Ti1Zr1	BCC	C	AC	25.0	1600.0
Hf1Mo1Ta1Ti1Zr1	BCC	C	AC	800.0	1045.0
Hf1Mo1Ta1Ti1Zr1	BCC	C	AC	1000.0	855.0
Hf1Mo1Ta1Ti1Zr1	BCC	C	AC	1200.0	404.0
Hf1Nb1Ta1Ti1	BCC	C	A	25.0	847.0
Hf1Nb1Ta1Ti1	BCC	C	A	200.0	610.0
Hf1Nb1Ta1Ti1	BCC	C	A	600.0	473.0
Hf1Nb1Ta1Ti1Zr1	BCC	C	OTHER	25.0	929.0
Hf1Nb1Ta1Ti1Zr1	BCC	C	OTHER	400.0	790.0
Hf1Nb1Ta1Ti1Zr1	BCC	C	OTHER	600.0	675.0
Hf1Nb1Ta1Ti1Zr1	BCC	C	OTHER	800.0	535.0
Hf1Nb1Ta1Ti1Zr1	BCC	C	OTHER	1000.0	295.0
Hf1Nb1Ta1Ti1Zr1	BCC	C	OTHER	1200.0	92.0
Hf1Nb1Ta1Zr1	BCC	C	AC	25.0	1315.0
Hf1Nb1Ti1V1Zr1	BCC	C	A	25.0	1171.0
Hf1Nb1Ti1Zr1	BCC	C	AC	25.0	1000.0
Hf1Nb1Ti1Zr1	BCC	C	AC	800.0	303.0
Hf1Nb1Ti1Zr1	BCC	C	AC	1000.0	154.0
Mo0.1Nb1Ti1V0.3Zr1	BCC	C	A	25.0	932.0
Mo0.333Nb0.333Ti0.333V1Zr0.333	BCC	C	AC	25.0	1418.0
Mo0.3Nb1Ti1V0.3Zr1	BCC	C	A	25.0	1312.0
Mo0.3Nb1Ti1V1Zr1	BCC	C	A	25.0	1289.0
Mo0.5Nb0.5Ti0.5V1Zr0.5	BCC	C	AC	25.0	1538.0
Mo0.5Nb1Ti1V0.3Zr1	BCC	C	A	25.0	1301.0
Mo0.5Nb1Ti1V1Zr1	BCC	C	A	25.0	1473.0
Mo0.667Nb0.667Ti0.667V1Zr0.667	BCC	C	AC	25.0	1735.0
Mo0.7Nb1Ti1V0.3Zr1	BCC	C	A	25.0	1436.0
Mo0.7Nb1Ti1V1Zr1	BCC	C	A	25.0	1706.0
Mo1.3Nb1Ti1V0.3Zr1	BCC	C	AC	25.0	1603.0
Mo1.3Nb1Ti1V1Zr1	BCC	C	AC	25.0	1496.0
Mo1.5Nb1Ti1V0.3Zr1	BCC	C	AC	25.0	1576.0
Mo1Nb0.588Ti0.588V0.588Zr0.588	BCC	C	A	25.0	1645.0

Mo1Nb0.5Ti0.5V0.5Zr0.5	BCC	C	A	25.0	1765.0
Mo1Nb0.667Ti0.667V0.2Zr0.667	BCC	C	A	25.0	1576.0
Mo1Nb0.667Ti0.667V0.667Zr0.667	BCC	C	A	25.0	1603.0
Mo1Nb0.769Ti0.769V0.231Zr0.769	BCC	C	A	25.0	1603.0
Mo1Nb0.769Ti0.769V0.769Zr0.769	BCC	C	A	25.0	1496.0
Mo1Nb1Ta1Ti0.25W1	BCC	C	AC	25.0	1109.0
Mo1Nb1Ta1Ti0.5W1	BCC	C	AC	25.0	1211.0
Mo1Nb1Ta1Ti0.75W1	BCC	C	AC	25.0	1304.0
Mo1Nb1Ta1Ti1	BCC	C	A	25.0	1210.0
Mo1Nb1Ta1Ti1	BCC	C	A	60.0	1071.0
Mo1Nb1Ta1Ti1	BCC	C	A	100.0	1029.0
Mo1Nb1Ta1Ti1	BCC	C	A	200.0	868.0
Mo1Nb1Ta1Ti1	BCC	C	A	300.0	732.0
Mo1Nb1Ta1Ti1	BCC	C	A	400.0	685.0
Mo1Nb1Ta1Ti1	BCC	C	A	600.0	593.0
Mo1Nb1Ta1Ti1	BCC	C	A	800.0	564.0
Mo1Nb1Ta1Ti1	BCC	C	A	1000.0	539.0
Mo1Nb1Ta1Ti1V1	BCC	C	AC	25.0	1400.0
Mo1Nb1Ta1Ti1V1W1	BCC	C	AC	25.0	1515.0
Mo1Nb1Ta1Ti1V1W1	BCC	C	AC	600.0	973.0
Mo1Nb1Ta1Ti1V1W1	BCC	C	AC	800.0	791.3
Mo1Nb1Ta1Ti1V1W1	BCC	C	AC	1000.0	752.8666666666667
Mo1Nb1Ta1Ti1V1W1	BCC	C	AC	1200.0	659.0
Mo1Nb1Ta1Ti1W1	BCC	C	AC	25.0	1380.333333333333
Mo1Nb1Ta1Ti1W1	BCC	C	AC	600.0	689.0
Mo1Nb1Ta1Ti1W1	BCC	C	AC	800.0	674.0
Mo1Nb1Ta1Ti1W1	BCC	C	AC	1000.0	620.0
Mo1Nb1Ta1Ti1W1	BCC	C	AC	1200.0	586.0
Mo1Nb1Ta1V1	BCC	C	A	25.0	1525.0
Mo1Nb1Ta1V1W1	BCC	C	OTHER	25.0	1246.0
Mo1Nb1Ta1V1W1	BCC	C	OTHER	600.0	862.0
Mo1Nb1Ta1V1W1	BCC	C	OTHER	800.0	846.0
Mo1Nb1Ta1V1W1	BCC	C	OTHER	1000.0	842.0
Mo1Nb1Ta1V1W1	BCC	C	OTHER	1200.0	735.0
Mo1Nb1Ta1V1W1	BCC	C	OTHER	1400.0	656.0

Mo1Nb1Ta1V1W1	BCC	C	OTHER	1600.0	477.0
Mo1Nb1Ta1W1	BCC	C	AC	25.0	1027.0
Mo1Nb1Ta1W1	BCC	C	AC	600.0	561.0
Mo1Nb1Ta1W1	BCC	C	AC	800.0	552.0
Mo1Nb1Ta1W1	BCC	C	AC	1000.0	548.0
Mo1Nb1Ta1W1	BCC	C	AC	1200.0	506.0
Mo1Nb1Ta1W1	BCC	C	AC	1400.0	421.0
Mo1Nb1Ta1W1	BCC	C	AC	1600.0	405.0
Mo1Nb1Ti1	BCC	C	OTHER	25.0	1100.0
Mo1Nb1Ti1	BCC	C	OTHER	1000.0	504.0
Mo1Nb1Ti1	BCC	C	OTHER	1200.0	324.0
Mo1Nb1Ti1V0.25Zr1	BCC	C	AC	25.0	1776.0
Mo1Nb1Ti1V0.3Zr1	BCC	C	A	25.0	1455.0
Mo1Nb1Ti1V0.5Zr1	BCC	C	AC	25.0	1647.0
Mo1Nb1Ti1V0.75Zr1	BCC	C	AC	25.0	1708.0
Mo1Nb1Ti1V1	BCC	C	AC	25.0	1200.0
Mo1Nb1Ti1V1Zr1	BCC	C	A	25.0	1779.0
Mo1Nb1Ti1Zr1	BCC	C	AC	25.0	1592.0
Mo1Ta1Ti1V1	BCC	C	AC	25.0	1221.0
Nb0.5Ti0.5V1Zr0.5	BCC	C	OTHER	25.0	918.0
Nb0.5Ti0.5V1Zr0.5	BCC	C	OTHER	600.0	571.0
Nb0.5Ti0.5V1Zr0.5	BCC	C	OTHER	800.0	240.0
Nb0.5Ti0.5V1Zr0.5	BCC	C	OTHER	1000.0	72.0
Nb1Ta0.3Ti1Zr1	BCC	C	OTHER	25.0	882.0
Nb1Ta0.3Ti1Zr1	BCC	C	OTHER	1000.0	274.0
Nb1Ta0.3Ti1Zr1	BCC	C	OTHER	1200.0	102.0
Nb1Ta1Ti1	BCC	C	OTHER	25.0	573.0
Nb1Ta1Ti1	BCC	C	OTHER	100.0	486.0
Nb1Ta1Ti1	BCC	C	OTHER	200.0	378.0
Nb1Ta1Ti1	BCC	C	OTHER	300.0	314.0
Nb1Ta1Ti1	BCC	C	OTHER	400.0	232.0
Nb1Ta1Ti1	BCC	C	OTHER	600.0	222.0
Nb1Ta1Ti1	BCC	C	OTHER	800.0	210.0
Nb1Ta1Ti1	BCC	C	OTHER	1000.0	160.0
Nb1Ta1Ti1V1	BCC	C	AC	25.0	1028.5

Nb1Ta1Ti1V1W1	BCC	C	AC	25.0	1420.0
Nb1Ta1Ti1W1	BCC	C	A	25.0	1054.0
Nb1Ta1Ti1W1	BCC	C	A	100.0	968.0
Nb1Ta1Ti1W1	BCC	C	A	200.0	869.0
Nb1Ta1Ti1W1	BCC	C	A	300.0	754.0
Nb1Ta1Ti1W1	BCC	C	A	400.0	627.0
Nb1Ta1Ti1W1	BCC	C	A	600.0	596.0
Nb1Ta1Ti1W1	BCC	C	A	800.0	564.0
Nb1Ta1Ti1W1	BCC	C	A	1000.0	459.0
Nb1Ti1V0.3Zr1	BCC	C	A	25.0	866.0
Nb1Ti1V1Zr1	BCC	C	OTHER	25.0	1105.0
Nb1Ti1V1Zr1	BCC	C	OTHER	600.0	834.0
Nb1Ti1V1Zr1	BCC	C	OTHER	800.0	187.0
Nb1Ti1V1Zr1	BCC	C	OTHER	1000.0	58.0
Nb1Ti1Zr1	BCC	C	AC	25.0	1223.0
Nb1Ti1Zr1	BCC	C	AC	800.0	462.0
Nb1Ti1Zr1	BCC	C	AC	1000.0	218.0
Al1Nb1Ta0.5Ti1Zr0.5	B2	C	OTHER	25.0	1352.0
Al1Nb1Ta0.5Ti1Zr0.5	B2	C	OTHER	1000.0	535.0
Al1Nb1Ti1V1	B2	C	A	25.0	1000.0
Al1Nb1Ti1V1	B2	C	A	800.0	560.0
Al1Nb1Ti1V1	B2	C	A	600.0	780.0
Al0.25Nb1Ta1Ti1Zr1	BCC+B2	C	OTHER	25.0	1745.0
Al0.25Nb1Ta1Ti1Zr1	BCC+B2	C	OTHER	1000.0	366.0
Al0.3Nb1Ta1Ti1.4Zr1.3	BCC+B2	C	OTHER	25.0	1965.0
Al0.3Nb1Ta1Ti1.4Zr1.3	BCC+B2	C	OTHER	1000.0	236.0
Al0.3Nb1Ta1Ti1.4Zr1.3	BCC+B2	C	OTHER	800.0	362.0
Al0.5Nb1Ta0.8Ti1.5V0.2Zr1	BCC+B2	C	OTHER	25.0	2035.0
Al0.5Nb1Ta0.8Ti1.5V0.2Zr1	BCC+B2	C	OTHER	1000.0	220.0
Al0.5Nb1Ta0.8Ti1.5V0.2Zr1	BCC+B2	C	OTHER	800.0	796.0
Al1Mo0.5Nb1Ta0.5Ti1Zr1	BCC+B2	C	OTHER	1000.0	750.0
Al1Mo0.5Nb1Ta0.5Ti1Zr1	BCC+B2	C	OTHER	1200.0	250.0
Al1Nb1Ti1V1	B2	C	A	600.0	780.0
Al1Nb1Ti1V1	B2	C	A	800.0	560.0
Al1Nb1Ti1V1	B2	C	A	25.0	1000.0

Al1Mo0.5Nb1Ta0.5Ti1Zr0.5	B2	C	OTHER	1000.0	935.0
Al1Mo0.5Nb1Ta0.5Ti1Zr0.5	B2	C	OTHER	25.0	0.0
Al1Nb1Ta0.5Ti1Zr0.5	B2	C	OTHER	1000.0	535.0
Al1Nb1Ta0.5Ti1Zr0.5	B2	C	OTHER	25.0	1352.0
Al1Nb1Ti1Zr1	B2	C	AC	25.0	1509.0
Al0.25Cr0.5Fe1Mn0.625Ni0.375	B2+BCC	C	AC	25.0	994.0
Al0.35Cr0.5Fe1Mn0.625Ni0.375	B2+BCC	C	AC	25.0	942.0
Al0.25Cr0.5Fe1Mn0.625Ni0.375	B2+BCC	C	AC	400.0	943.0
Al0.35Cr0.5Fe1Mn0.625Ni0.375	B2+BCC	C	AC	400.0	833.0
Al0.25Cr0.5Fe1Mn0.625Ni0.375	B2+BCC	C	AC	600.0	359.0
Al0.35Cr0.5Fe1Mn0.625Ni0.375	B2+BCC	C	AC	600.0	326.0
Al1Co1Cr1Fe1Ni1	B2+BCC	C	AC	25.0	1382.3799999999999
Al1Cr1Fe1Ni1	BCC+B2	C	AC	25.0	1173.3999999999999
Al1Cr1Fe1Mo0.2Ni1	BCC+B2	C	AC	25.0	1487.0
Al1Cr1Fe1Mo0.5Ni1	BCC+B2	C	AC	25.0	1831.55
Al1Mo0.5Nb1Ta0.5Ti1Zr1	BCC+B2	C	OTHER	25.0	2000.0
Al1Mo0.5Nb1Ta0.5Ti1Zr1	BCC+B2	C	OTHER	800.0	1597.0
Al1Mo0.5Nb1Ta0.5Ti1Zr1	BCC+B2	C	OTHER	1000.0	745.0
Al1Mo0.5Nb1Ta0.5Ti1Zr1	BCC+B2	C	OTHER	1200.0	250.0
Al0.25Nb1Ta1Ti1Zr1	BCC+B2	C	OTHER	1000.0	366.0
Al0.25Nb1Ta1Ti1Zr1	BCC+B2	C	OTHER	25.0	1745.0
Al0.5Mo0.5Nb1Ta0.5Ti1Zr1	BCC+B2	C	OTHER	1000.0	579.0
Al0.5Mo0.5Nb1Ta0.5Ti1Zr1	BCC+B2	C	OTHER	25.0	2350.0
Al1Mo0.5Nb1Ta0.5Ti1Zr1	BCC+B2	C	OTHER	25.0	2197.0
Al0.885Cr1Fe1Ni1	BCC+B2	C	AC	25.0	770.0
Al1Cr0.889Fe0.889Ni0.889	BCC+B2	C	AC	25.0	1044.0
Al1Cr0.828Fe0.828Ni0.828	BCC+B2	C	AC	25.0	906.8
Al1Cr0.767Fe0.767Ni0.767	BCC+B2	C	AC	25.0	1122.8
Al0.5Cr0.5Fe1Ni1	BCC+B2	C	AC	25.0	780.0
Al1Co1Cr1Ni1	BCC+B2	C	AC	25.0	1750.0
Al1Co0.5Cr0.5Fe0.5Ni0.5	BCC+B2	C	AC	25.0	1241.0
Al1Co0.556Cr0.556Fe0.556Ni0.556Ti0.111	BCC+B2	C	AC	25.0	1324.0
Al1Co0.667Cr0.667Fe0.667Ni0.667Ti0.333	BCC+B2	C	AC	25.0	1566.0
Al1Co0.833Cr0.833Fe0.833Ni0.833Ti0.667	BCC+B2	C	AC	25.0	1503.0
Al1Co0.25Cr1Fe1Ni1	BCC+B2	C	AC	25.0	1363.0

Al1Co0.5Cr1Fe1Ni1	BCC+B2	C	AC	25.0	1349.0
Al1Co0.75Cr1Fe1Ni1	BCC+B2	C	AC	25.0	1331.0
Fe1Mo1Nb1Ti1V1	BCC+B2	C	AC	25.0	1707.0
Al0.5Cr1Fe1Mn1Ni1	BCC+B2	C	AC	25.0	1091.0
Al0.6Cr1Fe1Mn1Ni1	BCC+B2	C	AC	25.0	1130.0
Al0.7Cr1Fe1Mn1Ni1	BCC+B2	C	AC	25.0	1170.0
Al0.8Cr1Fe1Mn1Ni1	BCC+B2	C	AC	25.0	1200.0
Al1Cr1Fe1Mo0.5Ni1Ti0.25	BCC+B2	C	AC	25.0	2161.7
Al1Cr1Fe1Mo0.5Ni1Ti0.4	BCC+B2	C	AC	25.0	2185.1
Al1Cr1Fe1Mo0.5Ni1Ti0.5	BCC+B2	C	AC	25.0	2228.7
Al1Cr1Fe1Mo0.5Ni1Ti0.6	BCC+B2	C	AC	25.0	1314.5

Table S6. Dataset comprising Plasticity values (%) for BCC & BCC+B2 HEAs. The dataset includes columns for alloy composition, phase constitution, mechanical testing method, processing condition, testing temperature (°C), and corresponding yield strength values. In this context, "A" denotes alloys that have undergone Annealing, "AC" represents those in the As Cast condition, and "OTHER" refers to all other processing conditions. The mechanical testing method is specified as "C" for Compression testing.

**BCC & BCC+B2 Plasticity Dataset**					
**Alloy**	**Phases**	**Mechanical Testing**	**Processing Condition**	**Test Temperature C**	**Plasticity (%)**
Al1Co1Cr1Fe1Ni1	BCC+B2	C	AC	25	23.0
Al1Co1Cr1Fe1Ni1	BCC+B2	C	AC	25	16.0
Al1Co1Cr1Fe1Ni1	BCC+B2	C	AC	25	23.0
Al0.25Nb1Ta1Ti1Zr1	BCC+B2	C	OTHER	1000	2.0
Al0.5Cr0.5Fe1Ni1	BCC+B2	C	AC	25	17.0
Al0.5Cr1Fe1Mn1Ni1	BCC+B2	C	AC	25	50.0
Al0.5Mo0.5Nb1Ta0.5Ti1Zr1	BCC+B2	C	OTHER	1000	2.0
Al0.6Cr1Fe1Mn1Ni1	BCC+B2	C	AC	25	53.0
Al0.7Cr1Fe1Mn1Ni1	BCC+B2	C	AC	25	55.0
Al0.885Cr1Fe1Ni1	BCC+B2	C	AC	25	37.1
Al0.8Cr1Fe1Mn1Ni1	BCC+B2	C	AC	25	55.0
Al1Co0.25Cr1Fe1Ni1	BCC+B2	C	AC	25	19.0
Al1Co0.556Cr0.556Fe0.556Ni0.556Ti0.111	BCC+B2	C	AC	25	10.02
Al1Co0.5Cr0.5Fe0.5Ni0.5	BCC+B2	C	AC	25	9.98

Al1Co0.5Cr1Fe1Ni1	BCC+B2	C	AC	25	20.0
Al1Co0.667Cr0.667Fe0.667Ni0.667Ti0.333	BCC+B2	C	AC	25	11.85
Al1Co0.75Cr1Fe1Ni1	BCC+B2	C	AC	25	20.0
Al1Co0.833Cr0.833Fe0.833Ni0.833Ti0.667	BCC+B2	C	AC	25	7.73
Al1Co1Cr1Fe1Ni1	BCC+B2	C	AC	25	10.0
Al1Co1Cr1Fe1Ni1	BCC+B2	C	AC	25	10.0
Al1Co1Cr1Fe1Ni1	BCC+B2	C	AC	25	22.7
Al1Co1Cr1Ni1	BCC+B2	C	AC	25	16.7
Al1Cr0.767Fe0.767Ni0.767	BCC+B2	C	AC	25	24.7
Al1Cr0.828Fe0.828Ni0.828	BCC+B2	C	AC	25	31.4
Al1Cr0.889Fe0.889Ni0.889	BCC+B2	C	AC	25	27.1
Al1Cr1Fe1Ni1	BCC+B2	C	AC	25	34.6
Al1Mo0.5Nb1Ta0.5Ti1Zr1	BCC+B2	C	OTHER	25	10.0
Al1Mo0.5Nb1Ta0.5Ti1Zr1	BCC+B2	C	OTHER	800	11.0
Al1Mo0.5Nb1Ta0.5Ti1Zr1	BCC+B2	C	OTHER	1000	50.0
Al1Mo0.5Nb1Ta0.5Ti1Zr1	BCC+B2	C	OTHER	1200	50.0
Al1Mo0.5Nb1Ta0.5Ti1Zr1	BCC+B2	C	OTHER	25	10.0
Al1Mo0.5Nb1Ta0.5Ti1Zr1	BCC+B2	C	OTHER	800	11.0
Al1Mo0.5Nb1Ta0.5Ti1Zr1	BCC+B2	C	OTHER	1000	50.0
Al1Mo0.5Nb1Ta0.5Ti1Zr1	BCC+B2	C	OTHER	1200	50.0
Fe1Mo1Nb1Ti1V1	BCC+B2	C	AC	25	1.7
Al1Nb1Ti1Zr1	B2	C	AC	25	8.6
Al1Mo0.5Nb1Ta0.5Ti1Zr0.5	B2	C	OTHER	1000	2.0
Al1Nb1Ta0.5Ti1Zr0.5	B2	C	OTHER	1000	1.0
Al1Nb1Ti1V1	B2	C	A	600	14.0
Al1Nb1Ti1V1	B2	C	A	800	50.0
Al1Nb1Ti1V1	B2	C	A	25	6.0
Al0.214Nb0.714Ta0.571Ti1V0.143Zr0.929	BCC	C	OTHER	25	5.0
Al0.214Nb0.714Ta0.714Ti1Zr0.929	BCC	C	OTHER	25	5.0
Al0.25Co1Cr1Cu1Fe1Mn1Ni1Ti1V1	BCC	C	AC	25	2.0
Al0.25Mo1Nb1Ti1V1	BCC	C	AC	25	13.0
Al0.2Mo1Ta1Ti1V1	BCC	C	AC	25	7.0
Al0.333Nb0.667Ta0.533Ti1V0.133Zr0.667	BCC	C	OTHER	25	5.0
Al0.3Hf1Nb1Ta1Ti1Zr1	BCC	C	AC	25	50.0
Al0.3Nb1Ta0.8Ti1.4V0.2Zr1.3	BCC	C	AC	25	5.0

Al0.3Nb1Ta0.8Ti1.4V0.2Zr1.3	BCC	C	AC	800	50.0
Al0.3Nb1Ta0.8Ti1.4V0.2Zr1.3	BCC	C	AC	1000	50.0
Al0.3Nb1Ta1Ti1.4Zr1.3	BCC	C	AC	25	5.0
Al0.3Nb1Ta1Ti1.4Zr1.3	BCC	C	AC	800	50.0
Al0.3Nb1Ta1Ti1.4Zr1.3	BCC	C	AC	1000	50.0
Al0.4Hf0.6Nb1Ta1Ti1Zr1	BCC	C	AC	25	10.0
Al0.4Hf0.6Nb1Ta1Ti1Zr1	BCC	C	AC	800	50.0
Al0.4Hf0.6Nb1Ta1Ti1Zr1	BCC	C	AC	1000	50.0
Al0.5Hf1Nb1Ta1Ti1Zr1	BCC	C	AC	25	46.0
Al0.5Mo1Nb1Ti1V1	BCC	C	AC	25	11.0
Al0.5Nb1Ta0.8Ti1.5V0.2Zr1	BCC	C	AC	25	4.5
Al0.5Nb1Ta0.8Ti1.5V0.2Zr1	BCC	C	AC	800	50.0
Al0.5Nb1Ta0.8Ti1.5V0.2Zr1	BCC	C	AC	1000	50.0
Al0.667Nb1Ta0.333Ti1Zr0.333	BCC	C	OTHER	25	4.0
Al0.667Nb1Ta0.333Ti1Zr0.333	BCC	C	OTHER	800	12.0
Al0.6Mo1Ta1Ti1V1	BCC	C	AC	25	4.0
Al0.75Hf1Nb1Ta1Ti1Zr1	BCC	C	AC	25	30.0
Al0.75Mo1Nb1Ti1V1	BCC	C	AC	25	8.0
Al1.5Co1Cr1Fe1Ni1Ti1	BCC	C	AC	25	10.0
Al1Co1Cr1Cu1Ni1Ti1	BCC	C	AC	25	8.0
Al1Co1Cr1Fe1Mo0.1Ni1	BCC	C	AC	25	9.0
Al1Co1Cr1Fe1Nb0.1Ni1	BCC	C	AC	25	17.0
Al1Co1Cr1Fe1Ni1	BCC	C	AC	25	25.0
Al1Co1Cr1Fe1Ni1Ti0.5	BCC	C	AC	25	23.0
Al1Co1Cr1Fe1Ni1Ti1	BCC	C	AC	25	9.0
Al1Cr0.5Nb1Ti1V1	BCC	C	A	25	0.8
Al1Cr0.5Nb1Ti1V1	BCC	C	A	600	2.5
Al1Cr1Fe1Mo0.2Ni1	BCC	C	AC	25	29.0
Al1Cr1Fe1Mo0.5Ni1	BCC	C	AC	25	13.0
Al1Cr1Fe1Ni1	BCC	C	AC	25	29.0
Al1Cr1Mo1Nb1Ti1	BCC	C	AC	400	2.0
Al1Cr1Mo1Nb1Ti1	BCC	C	AC	600	3.0
Al1Cr1Mo1Nb1Ti1	BCC	C	AC	800	2.0
Al1Cr1Mo1Nb1Ti1	BCC	C	AC	1000	15.0
Al1Cr1Mo1Nb1Ti1	BCC	C	AC	1200	24.0

Al1Mo0.5Nb1Ta0.5Ti1Zr1	BCC	C	AC	25	10.0
Al1Mo1Nb1Ti1V1	BCC	C	AC	25	3.0
Al1Nb1.5Ta0.5Ti1.5Zr0.5	BCC	C	AC	25	4.0
Al1Nb1Ti1V1	BCC	C	A	25	5.1
Al1Nb1Ti1V1	BCC	C	A	600	12.25
Al2Co1Cr1Cu1Fe1Mn1Ni1Ti1V1	BCC	C	AC	25	2.0
Al2Co1Cr1Fe1Ni1Ti1	BCC	C	AC	25	5.0
Co1Cr1Mo1Nb1Ti0.4	BCC	C	AC	25	5.0
Cr1Fe1Ni1Ti0.4	BCC	C	AC	25	6.5
Hf0.24Nb0.23Ti0.38V0.15	BCC	C	AC	25	20.6
Hf0.5Mo0.5Nb1Ti1Zr1	BCC	C	AC	25	25.0
Hf0.5Mo1Nb1Ti1Zr1	BCC	C	AC	25	12.09
Hf0.5Nb0.5Ta0.5Ti1.5Zr1	BCC	C	AC	25	18.8
Hf1.5Mo1Nb1Ti1Zr1	BCC	C	AC	25	16.83
Hf1Mo0.25Nb1Ta1Ti1Zr1	BCC	C	AC	25	50.0
Hf1Mo0.5Nb1Ta1Ti1Zr1	BCC	C	AC	25	50.0
Hf1Mo0.5Nb1Ti1V0.5	BCC	C	AC	25	35.0
Hf1Mo0.5Nb1Ti1V0.5	BCC	C	AC	1000	35.0
Hf1Mo0.5Nb1Ti1V0.5	BCC	C	AC	1200	35.0
Hf1Mo0.75Nb1Ta1Ti1Zr1	BCC	C	AC	25	50.0
Hf1Mo1.5Nb1Ti1Zr1	BCC	C	AC	25	10.83
Hf1Mo1Nb0.5Ti1Zr1	BCC	C	AC	25	13.02
Hf1Mo1Nb1.5Ti1Zr1	BCC	C	AC	25	23.97
Hf1Mo1Nb1Ta1Ti1	BCC	C	AC	25	27.0
Hf1Mo1Nb1Ta1Ti1Zr1	BCC	C	AC	25	12.0
Hf1Mo1Nb1Ta1Ti1Zr1	BCC	C	AC	800	23.0
Hf1Mo1Nb1Ta1Ti1Zr1	BCC	C	AC	1000	30.0
Hf1Mo1Nb1Ta1Ti1Zr1	BCC	C	AC	1200	30.0
Hf1Mo1Nb1Ti0.5Zr1	BCC	C	AC	25	12.08
Hf1Mo1Nb1Ti1.5Zr1	BCC	C	AC	25	28.98
Hf1Mo1Nb1Ti1Zr0.5	BCC	C	AC	25	18.02
Hf1Mo1Nb1Ti1Zr1	BCC	C	AC	25	10.12
Hf1Mo1Nb1Ti1Zr1.5	BCC	C	AC	25	16.09
Hf1Mo1Ta1Ti1Zr1	BCC	C	AC	25	4.0
Hf1Mo1Ta1Ti1Zr1	BCC	C	AC	800	19.0

Hf1Mo1Ta1Ti1Zr1	BCC	C	AC	1000	30.0
Hf1Mo1Ta1Ti1Zr1	BCC	C	AC	1200	30.0
Hf1Nb1Ta1Ti1Zr1	BCC	C	AC	25	50.0
Hf1Nb1Ta1Zr1	BCC	C	AC	25	21.6
Hf1Nb1Ti1V1Zr1	BCC	C	AC	25	29.6
Hf1Nb1Ti1Zr1	BCC	C	AC	25	52.0
Hf1Nb1Ti1Zr1	BCC	C	AC	800	51.0
Hf1Nb1Ti1Zr1	BCC	C	AC	1000	51.0
Mo0.1Nb1Ti1V0.3	BCC	C	AC	25	45.0
Mo0.1Nb1Ti1V0.3Zr1	BCC	C	AC	25	45.0
Mo0.333Nb0.333Ti0.333V1Zr0.333	BCC	C	AC	25	24.0
Mo0.3Nb1Ti1V0.3	BCC	C	AC	25	50.0
Mo0.3Nb1Ti1V0.3Zr1	BCC	C	AC	25	50.0
Mo0.3Nb1Ti1V1Zr1	BCC	C	AC	25	42.0
Mo0.5Nb0.5Ti0.5V1Zr0.5	BCC	C	AC	25	23.0
Mo0.5Nb1Ti1V0.3	BCC	C	AC	25	43.0
Mo0.5Nb1Ti1V0.3Zr1	BCC	C	AC	25	43.0
Mo0.5Nb1Ti1V1Zr1	BCC	C	AC	25	32.0
Mo0.667Nb0.667Ti0.667V1Zr0.667	BCC	C	AC	25	20.0
Mo0.7Nb1Ti1V0.3	BCC	C	AC	25	27.0
Mo0.7Nb1Ti1V0.3Zr1	BCC	C	AC	25	26.6
Mo0.7Nb1Ti1V1Zr1	BCC	C	AC	25	32.0
Mo1.0Nb1Ti1V0.3Zr1	BCC	C	AC	25	25.0
Mo1.3Nb1Ti1V0.3	BCC	C	AC	25	20.0
Mo1.3Nb1Ti1V0.3Zr1	BCC	C	AC	25	20.0
Mo1.3Nb1Ti1V1Zr1	BCC	C	AC	25	30.0
Mo1.3Nb1Ti1V1Zr1	BCC	C	AC	25	30.0
Mo1.5Nb1Ti1V0.3	BCC	C	AC	25	8.0
Mo1.5Nb1Ti1V0.3Zr1	BCC	C	AC	25	8.0
Mo1.5Nb1Ti1V1Zr1	BCC	C	AC	25	20.0
Mo1.7Nb1Ti1V1Zr1	BCC	C	AC	25	15.0
Mo1Nb1Ta1Ti0.25W1	BCC	C	AC	25	2.5
Mo1Nb1Ta1Ti0.5W1	BCC	C	AC	25	5.9
Mo1Nb1Ta1Ti0.75W1	BCC	C	AC	25	8.4
Mo1Nb1Ta1Ti1V1	BCC	C	AC	25	30.0

Mo1Nb1Ta1Ti1V1W1	BCC	C	AC	25	10.6
Mo1Nb1Ta1Ti1W1	BCC	C	AC	25	14.1
Mo1Nb1Ta1V1	BCC	C	A	25	21.0
Mo1Nb1Ta1V1W1	BCC	C	AC	25	2.0
Mo1Nb1Ta1V1W1	BCC	C	AC	600	13.0
Mo1Nb1Ta1V1W1	BCC	C	AC	800	17.0
Mo1Nb1Ta1V1W1	BCC	C	AC	1000	19.0
Mo1Nb1Ta1V1W1	BCC	C	AC	1200	7.5
Mo1Nb1Ta1V1W1	BCC	C	AC	1400	40.0
Mo1Nb1Ta1V1W1	BCC	C	AC	1600	40.0
Mo1Nb1Ta1W1	BCC	C	AC	25	2.0
Mo1Nb1Ta1W1	BCC	C	AC	600	40.0
Mo1Nb1Ta1W1	BCC	C	AC	800	40.0
Mo1Nb1Ta1W1	BCC	C	AC	1000	40.0
Mo1Nb1Ta1W1	BCC	C	AC	1200	40.0
Mo1Nb1Ta1W1	BCC	C	AC	1400	40.0
Mo1Nb1Ta1W1	BCC	C	AC	1600	40.0
Mo1Nb1Ta1W1	BCC	C	OTHER	25	7.5
Mo1Nb1Ti1V0.25Zr1	BCC	C	AC	25	30.0
Mo1Nb1Ti1V0.3	BCC	C	AC	25	25.0
Mo1Nb1Ti1V0.5Zr1	BCC	C	AC	25	28.0
Mo1Nb1Ti1V0.75Zr1	BCC	C	AC	25	29.0
Mo1Nb1Ti1V1	BCC	C	AC	25	26.0
Mo1Nb1Ti1V1.5Zr1	BCC	C	AC	25	20.0
Mo1Nb1Ti1V1Zr1	BCC	C	AC	25	26.0
Mo1Nb1Ti1V2Zr1	BCC	C	AC	25	23.0
Mo1Nb1Ti1V3Zr1	BCC	C	AC	25	24.0
Mo1Nb1Ti1Zr1	BCC	C	AC	25	34.0
Mo1Ta1Ti1V1	BCC	C	AC	25	10.0
Mo2Nb1Ti1V1Zr1	BCC	C	AC	25	12.0
Nb1Ta1Ti1V1	BCC	C	OTHER	25	28.566666666666667
Nb1Ta1Ti1V1W1	BCC	C	AC	25	20.0
Nb1Ta1V1W1	BCC	C	AC	25	12.0
Nb1Ta1V1W1	BCC	C	AC	25	12.0
Nb1Ti1V0.3Zr1	BCC	C	AC	25	45.0

Nb1Ti1V1Zr1	BCC	C	AC	25	50.0
Nb1Ti1V2Zr1	BCC	C	AC	25	50.0
Nb1Ti1Zr1	BCC	C	AC	25	48.0
Nb1Ti1Zr1	BCC	C	AC	800	51.0
Nb1Ti1Zr1	BCC	C	AC	1000	51.0
